# Efficacy and Safety of Concurrent Chemoradiotherapy as First‐Line Treatment for Stage IVB Cervical Cancer: A Single‐Center Retrospective Observational Study

**DOI:** 10.1111/jog.70119

**Published:** 2025-10-29

**Authors:** Yumi Ishidera, Takayoshi Iijima, Masahiro Aichi, Yuki Ogawara, Yuichi Imai, Madoka Sugiura, Masaharu Hata, Etsuko Miyagi, Taichi Mizushima

**Affiliations:** ^1^ Department of Obstetrics and Gynecology Yokohama City University School of Medicine Graduate School of Medicine Yokohama Japan; ^2^ Department of Radiation Oncology Yokohama City University School of Medicine Graduate School of Medicine Yokohama Japan

**Keywords:** cervical cancer, chemotherapy initiation, concurrent chemoradiotherapy, distant metastasis, survival

## Abstract

**Aim:**

To evaluate the efficacy and safety of concurrent chemoradiotherapy prior to systemic chemotherapy in patients with stage IVB cervical cancer.

**Methods:**

This retrospective observational study included 40 patients diagnosed with stage IVB cervical cancer who received concurrent chemoradiotherapy as first‐line therapy at the Yokohama City University Hospital between 2007 and 2021. The evaluated outcomes included concurrent chemoradiotherapy response rate, chemotherapy initiation rate, adverse events, and overall survival.

**Results:**

The disease control rate of concurrent chemoradiotherapy was 72.5%, with no significant differences across the subgroups defined by the number of metastatic sites, presence of out‐of‐field lesions, parenchymal involvement, or histological subtype. Systemic chemotherapy was initiated in 89% of the patients, with a median interval of 39 days after concurrent chemoradiotherapy completion, except in one patient (3.6%) due to disease progression. Including recurrent cases, 91% of patients ultimately received systemic chemotherapy. Grade 3 or higher toxicity that significantly delayed chemotherapy initiation occurred in only one patient (3.6%). The median overall survival was 23 months, with no significant differences based on lesion distribution, parenchymal involvement, histological subtype, or metastatic burden.

**Conclusions:**

Concurrent chemoradiotherapy may be a feasible first‐line treatment option for stage IVB cervical cancer with manageable toxicity, acceptable disease control, and the potential to allow a timely transition to systemic chemotherapy.

## 1 Introduction

Although most cervical cancers are associated with human papillomavirus (HPV) infection and can be effectively prevented through HPV vaccination and screening, achieving comprehensive universal coverage remains challenging [1]. The standard treatment for FIGO stage IVB cervical cancer is systemic chemotherapy; however, the prognosis remains poor, with 5‐year overall survival (OS) rates of 19.4% in the United States (SEER, 2014–2020) [2] and 25.0% in Japan, among patients who initiated treatment in 2017 [3].

Recent studies have explored the use of targeted immunotherapeutic agents to address this unmet clinical requirement. The combination of systemic chemotherapy with either bevacizumab (GOG‐240 trial [4]) or pembrolizumab (KEYNOTE‐826 trial [5]) has shown significant improvements in the clinical outcomes of patients with advanced or recurrent cervical cancer. Nonetheless, the OS remains suboptimal.

Accumulating evidence suggests that definitive pelvic radiotherapy may enhance the clinical outcomes in patients with cervical cancer and distant metastases. Concurrent chemoradiotherapy (CCRT) has demonstrated efficacy, particularly in cases of oligometastatic disease [6], distant lymph node involvement [7, 8], and bone and pulmonary metastases [9, 10]. Nonetheless, the optimal timing and sequencing of radiotherapy remain undefined, as many studies have included heterogeneous protocols involving neoadjuvant or adjuvant chemotherapy [11], or have lacked clarity regarding treatment sequencing [12, 13, 14].

Among the various approaches, administering CCRT before systemic chemotherapy may offer the advantage of improved local control of the primary tumor and a reduction in tumor‐related hemorrhage. However, delaying systemic chemotherapy raises concerns regarding the potential progression of distant metastases, particularly those involving parenchymal organs.

Therefore, we conducted a retrospective study to evaluate the efficacy and safety of initiating treatment with CCRT in patients with FIGO stage IVB cervical cancer, including those with parenchymal metastases.

## 2 Methods

This retrospective observational study was conducted using the medical records of patients diagnosed with stage IVB cervical cancer at Yokohama City University Hospital between January 1, 2007, and December 31, 2021. Eligible patients were aged 18 years or older and had received concurrent chemoradiotherapy (CCRT) as an initial treatment strategy. Staging was reclassified according to the 2018 International Federation of Gynecology and Obstetrics (FIGO) guidelines. All pathologically confirmed histological subtypes were included. Patients with active synchronous malignancies were excluded.

The following data were collected for each patient: age, T and N stages at diagnosis according to the Union for International Cancer Control (UICC) 8th edition, number and sites of distant metastases, histological subtype, pelvic radiation dose, use and dose of intracavitary brachytherapy, use of radiation for distant metastases, concurrent chemotherapy regimen, treatment response, treatment‐related adverse events, and survival outcomes. Oligometastatic disease was defined as the presence of one to five metastatic lesions [15].

Treatment responses were assessed based on the Response Evaluation Criteria in Solid Tumors (RECIST) version 1.1 [16], and categorized as complete response (CR), partial response (PR), stable disease (SD), progressive disease (PD), and disease control rate (DCR). Treatment‐related adverse events were evaluated using the Common Terminology Criteria for Adverse Events (CTCAE) version 5.0 [17]. Early adverse events were defined as those occurring within 90 days of CCRT completion, whereas late adverse events were defined as those occurring beyond 90 days. Late adverse events (AEs) were specifically evaluated for urinary and gastrointestinal disorders.

Statistical analyses were performed to evaluate treatment efficacy, overall survival (OS; from treatment initiation to death), and progression‐free survival (PFS; from treatment initiation to disease progression). Treatment response rates were compared using the chi‐square test according to the presence of out‐of‐radiation field lesions, parenchymal metastases, histological subtypes, and the number of metastases. Survival curves were estimated using the Kaplan–Meier method, and comparisons between groups were made using the log‐rank test.

This study was approved by the Ethics Committee of Yokohama City University (Approval No. F240400003). Informed consent was waived by the ethics committee due to the retrospective nature of the study and the use of anonymized data.

## 3 Results

### 3.1 Patients Clinicopathological Characteristics

Forty patients with FIGO stage IVB cervical cancer who received definitive CCRT between 2007 and 2021 were analyzed (Table 1). The median follow‐up period was 16.5 months (range, 3–145 months). The median patient age was 57 years, and the predominant histologies were squamous cell carcinoma (62.5%) and adenocarcinoma (27.5%). Oligometastatic disease (≤ 5 lesions) was present in 65.0% of patients, with the lungs, supraclavicular nodes, and bones being the most common metastatic sites. Distant metastases were observed in 42.5% of cases. All patients received external beam radiation therapy with three‐dimensional conformal radiation therapy (3D‐CRT), with a median total dose of 50.2 Gy (range, 40.0–61.2 Gy). Central shielding was generally applied; however, it was omitted in cases where tumor localization, the degree of tumor shrinkage, or difficulty with tandem insertion precluded its use. Intracavitary brachytherapy was administered in 36 of 40 patients (90%). In cases where anatomical limitations existed or patient consent could not be obtained, brachytherapy was not performed. Concurrent chemotherapy consisted of cisplatin at 40 mg/m^2^ weekly, with a median of 5 cycles (range, 2–7). In 6 patients with renal dysfunction who were not candidates for cisplatin, concurrent chemoradiotherapy was performed using paclitaxel (135 mg/m^2^, day 1) plus carboplatin (AUC 5, day 1), administered every 3 weeks for a median of 2 cycles (range, 2–3).

**TABLE 1:** Patient demographics, clinical characteristics, and treatment details.

Age, median (range), years	57 (39–82)
ECOG performance status, *n* (%)	
0	14 (35.0)
1	18 (45.0)
2	8 (20.0)
Histology, *n* (%)	
Squamous cell carcinoma	25 (62.5)
Adenocarcinoma	11 (27.5)
Adenosquamous carcinoma	2 (5.0)
Other	2 (5.0)
Tumor stage, *n* (%)	
T1	1 (2.5)
T2	5 (12.5)
T3	29 (72.5)
T4	5 (12.5)
Nodal stage, *n* (%)	
N0	7 (17.5)
N1	33 (82.5)
Primary tumor size, median (range), cm	63.5 (25–128)
Number of metastatic sites, *n* (%)	
1	9 (22.5)
2–5	17 (42.5)
≧ 6	14 (35.0)
Metastatic sites, *n* (%)	
Parenchymal metastases	
Lung	15 (37.5)
Bone	11 (27.5)
Liver	5 (12.5)
Peritoneum	2 (5.0)
Pleura	1 (2.5)
Lymphatic metastases	
Supraclavicular lymph node	15 (37.5)
Inguinal lymph node	6 (15.0)
Mediastinal lymph node	4 (10.0)
Other	3 (7.5)
Radiotherapy	
EBRT dose, median (range), Gy	50.4 (40.0–61.2)
Brachytherapy, *n* (%)	36 (90.0)
Extra pelvic radiation therapy, *n* (%)	17 (42.5)
Concurrent chemotherapy, *n* (%)	
Cisplatin	34 (85.0)
Carboplatin/paclitaxel	6 (15.0)

Abbreviations: ECOG: eastern cooperative oncology group, EBRT: external beam radiotherapy.

### 3.2 Effectiveness of CCRT According to Clinicopathological Factors

Among the 40 patients treated with the initial CCRT, 27.5% achieved CR, 35.0% achieved PR, 10.0% achieved SD, and 27.5% achieved PD, with a DCR of 72.5% (Table 2). The DCR was not significantly associated with the number of metastases, the presence of out‐of‐field lesions, visceral involvement, or histology. CR was significantly more frequent in patients with oligometastatic disease than in those with polymetastatic disease (38.5% vs. 7.1%, *p* = 0.034), in those without out‐of‐field lesions (46.7% vs. 16.0%, *p* = 0.035), and in those with nodal metastases than in those with visceral metastases (57.1% vs. 11.5%, *p* = 0.002). Four patients achieved CR despite having lesions outside the radiation field: solitary 4‐mm pulmonary metastases, 5‐mm pulmonary oligometastatic lesions, 14‐mm supraclavicular lymph node oligometastatic lesions, and 28‐mm pulmonary polymetastatic lesions. All 11 cases that resulted in PD following CCRT showed disease progression to non‐irradiated distant sites, including the lungs (*n* = 7), bones (*n* = 3), liver (*n* = 2), peritoneum (*n* = 1), and hilar lymph nodes (*n* = 1).

**TABLE 2:** Disease control rate and complete response according to clinical factors.

	DCR		CR	
	% (*n*)	*p*	% (*n*)	*p*
All patients	72.5 (29/40)		27.5 (11/40)	
Number of metastatic sites				
1–5	76.9 (20/26)	0.393	38.5 (10/26)	0.034*
≧ 6	64.3 (9/14)		7.1 (1/14)	
Lesions outside the radiation field				
Yes	72.0 (18/25)	0.927	16.0 (4/25)	0.035*
No	73.3 (11/15)		46.7 (7/15)	
Parenchymal metastases				
Yes	69.2 (18/26)	0.528	11.5 (3/26)	0.002*
No	78.6 (11/14)		57.1 (8/14)	
Histology				
SCC	68.0 (17/25)	0.410	36.0 (9/25)	0.120
Other	80.0 (12/15)		13.3 (2/15)	

Abbreviations: CR: complete response, DCR: disease control rate, SCC: squamous cell carcinoma.

* *p* < 0.05.

### 3.3 Secondary Treatment After CCRT

**FIGURE 1:**
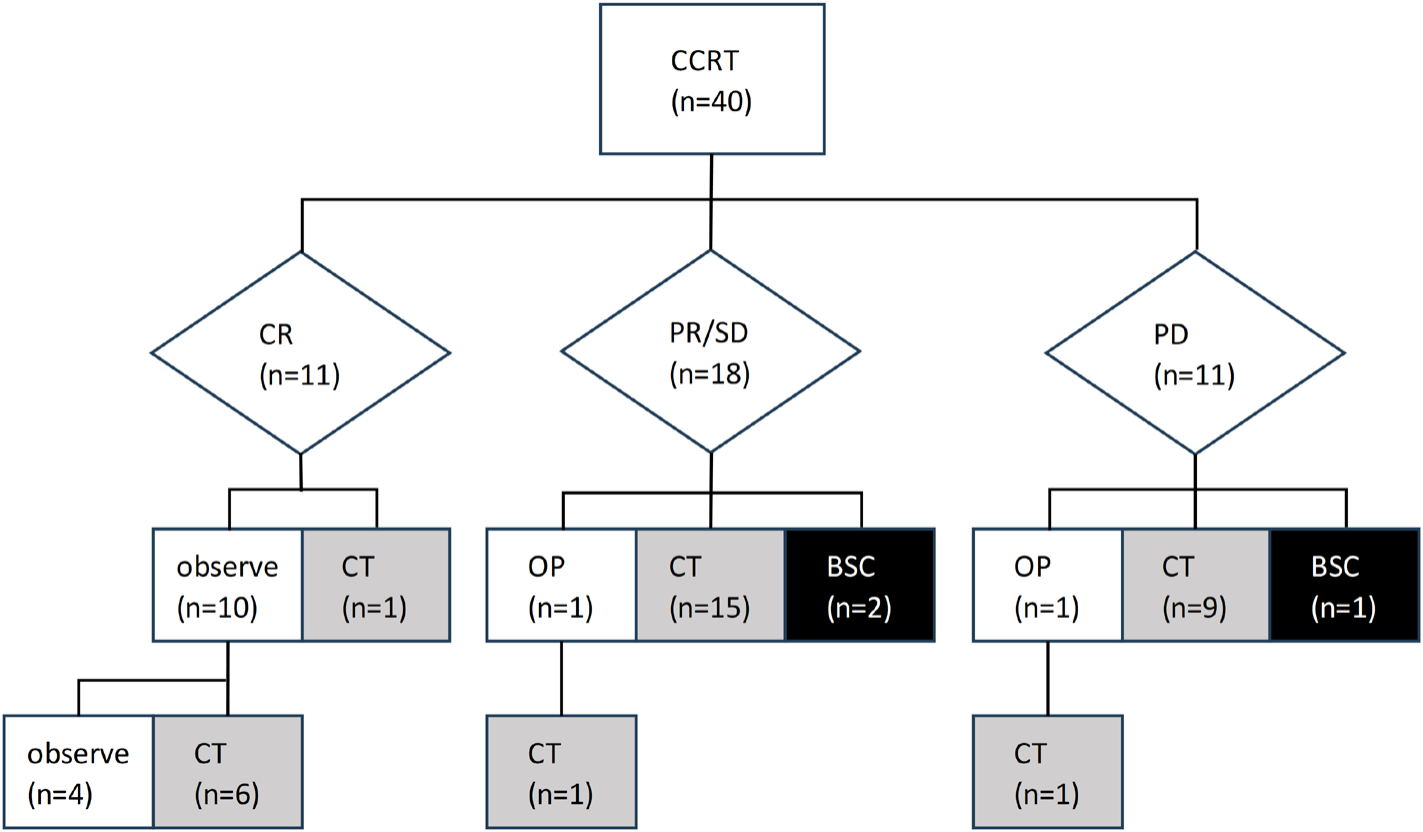
Initial response after CCRT and secondary treatment. BSC, best supportive care; CCRT, concurrent chemoradiotherapy; CR, complete response; CT, chemotherapy; OP, operation; PD, progressive disease; PR, partial response; SD, stable disease.

Figure 1 illustrates the treatment responses after CCRT and subsequent secondary therapies. Observation was selected for 10 patients who achieved CR after initial CCRT, one patient with PR followed by hysterectomy, and one patient with PD followed by lung resection. The remaining 28 patients were candidates for systemic chemotherapy; 89% (25/28) received chemotherapy, and 11% (3/28) selected best supportive care (BSC). In 25 patients who initiated chemotherapy, the median interval from CCRT completion to chemotherapy was 39 days (range, 1–141). The timing of chemotherapy initiation was not standardized but rather determined at the discretion of the treating physician, based on each patient's condition and recovery status after CCRT. In one case of Grade 3 enterocolitis, chemotherapy initiation was delayed by 141 days.

Among the 12 patients under observation, 10 were CR patients and 2 were surgical cases; 8 developed recurrence during follow‐up and underwent chemotherapy. The median time to chemotherapy in these patients was 256 days (range, 112–1053). Recurrences were in‐field (pelvis, supraclavicular lymph nodes) in 42.9% (3/7) and out‐of‐field (lung, brain, supraclavicular lymph nodes) in 57.1% (4/7) of patients. Excluding the four patients who maintained CR, 91% (*n* = 33/36) of the remaining patients received systemic chemotherapy. The details of the chemotherapy regimens administered are summarized in Table S1. The number of regimens administered was one in 51.5% (17/33), two in 33.3% (11/33), and three or more in 15.2% (5/33).

Of the three patients who required chemotherapy but received BSC, two refused chemotherapy and one could not initiate chemotherapy due to disease progression. The patient initially presented with oligometastatic squamous cell carcinoma, developed peritoneal dissemination, and died of the disease 3 months after CCRT completion.

### 3.4 Frequency of Adverse Events

Table 3 summarizes early and late AEs of CTCAE Grade 3 or higher associated with CCRT. Among the early AEs, anemia and neutropenia were observed relatively frequently but recovered following treatment completion. In one patient who developed Grade 3 enterocolitis, the initiation of systemic chemotherapy was delayed by 141 days after CCRT completion. Regarding late AEs, hematuria was observed in 7.5% (3/40), urinary tract infection in 2.5% (1/40), fistula formation in 2.5% (1/40), lower gastrointestinal bleeding in 2.5% (1/40), diarrhea in 2.5% (1/40), and bowel obstruction in 2.5% (1/40). No treatment‐related deaths occurred.

**TABLE 3:** Incidence of grade ≥ 3 early and late adverse events.

Early adverse events	*n* (%)	Late adverse events	*n* (%)
Anemia	8 (20.0)	Hematuria	3 (7.5)
Leukopenia	9 (22.5)	Urinary tract infection	1 (2.5)
Neutropenia	8 (20.0)	Fistula	1 (2.5)
Thrombocytopenia	2 (5.0)	Lower gastrointestinal hemorrhage	1 (2.5)
Diarrhea	1 (2.5)	Diarrhea	1 (2.5)
Lower gastrointestinal hemorrhage	1 (2.5)	Ileus	1 (2.5)
Enterocolitis	1 (2.5)		
Urinary tract infection	1 (2.5)		
Anorexia	1 (2.5)		

*Note:* Early adverse events: within 90 days post‐CCRT; late adverse events: beyond 90 days.

### 3.5 Survival Analysis According to Clinicopathological Factors

**FIGURE 2:**
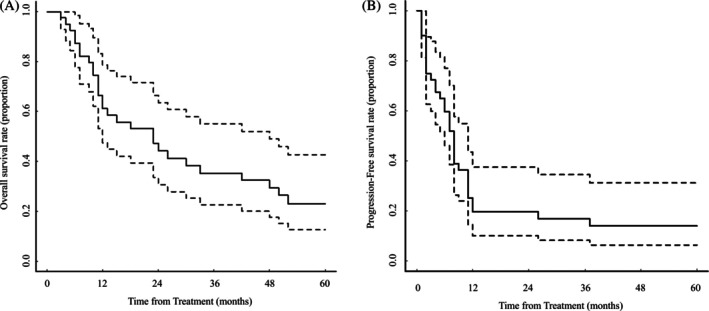
Kaplan–Meier curves of overall survival (OS) and progression‐free survival (PFS) in the overall population (A: OS, B: PFS).

**FIGURE 3:**
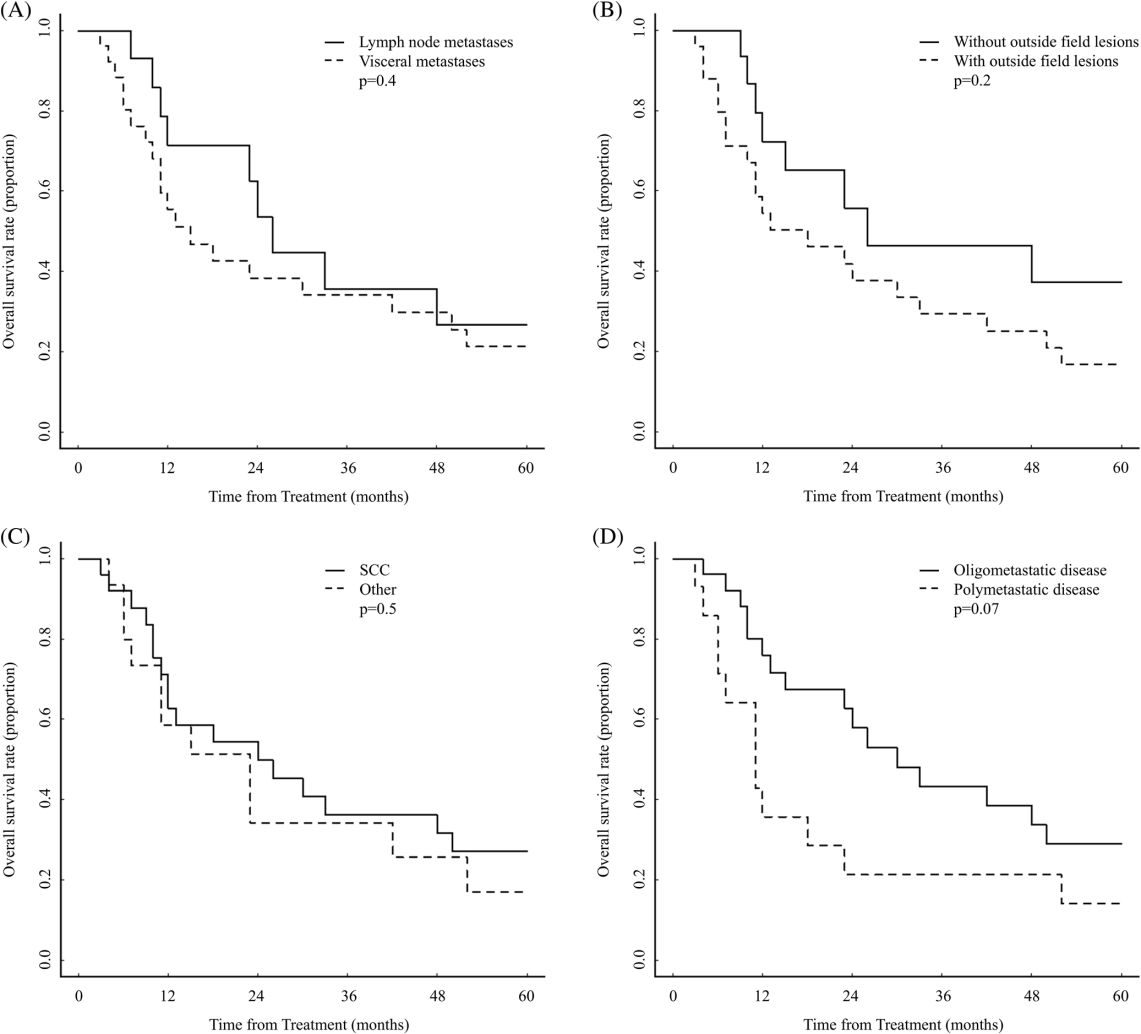
Kaplan–Meier curves of overall survival (OS) comparing the two groups. (A) lymph node metastases vs. visceral metastases; (B), without vs. with out‐of‐field lesions; (C), squamous cell carcinoma vs. other histological types; and (D), oligometastatic disease vs. polymetastatic disease. SCC, squamous cell carcinoma.

The median OS for the entire cohort (*n* = 40) was 23 months (95% CI, 12–48 months, Figure 2A), whereas the median PFS was 8 months (95% CI, 6–11 months, Figure 2B). No significant differences in OS or PFS were observed according to lesion distribution, parenchymal involvement, histological subtype, or metastatic burden. Comparisons of OS between the two groups are summarized in Figure 3A–D.

## 4 Discussion

### 4.1 Treatment Response to CCRT

This study aimed to evaluate the efficacy and safety of CCRT before systemic chemotherapy in patients with FIGO stage IVB cervical cancer. The disease control rate (DCR) after the initial CCRT was 72.5%, with consistent efficacy, regardless of the extent of metastasis, lesion location, or histology. Ultimately, 91% of the patients received systemic chemotherapy, including those with recurrence, supporting the feasibility of incorporating CCRT into multimodal treatment with systemic chemotherapy. Complete response (CR) was achieved in 27.5% of patients. Notably, 16% of the patients with out‐of‐field metastases achieved CR. Among these, three had oligometastatic disease with lesions < 20 mm, while one had multiple lung metastases exceeding six lesions and up to 28 mm in size. These responses may reflect the effects of concurrent chemotherapy or abscopal effects [18]. In support of this finding, Hasson et al. reported a 52% CR rate after CCRT in a predominantly oligometastatic cohort [11]. In our study, 8 of the 12 patients (66.7%) who achieved CR with CCRT or surgery experienced recurrence and required chemotherapy, with a median interval of 256 days. Similarly, Ning et al. reported progression in 55% of patients with ≤ 2 oligometastatic lesions after a median of 24.8 months, despite definitive radiotherapy [6]. These findings suggest that, while durable CR is achievable in selected cases, delaying systemic therapy through initial CCRT may provide a clinically meaningful treatment‐free interval. Therefore, CCRT may be a viable therapeutic option in patients with stage IVB cervical cancer.

### 4.2 Chemotherapy Administration After CCRT

In previously reported cohorts of patients with locally advanced cervical cancer, the administration rate of adjuvant systemic chemotherapy following cisplatin‐based CCRT ranged from 77% to 81%, with noncompliance being the primary reason for omission [19, 20]. In contrast, our study demonstrated a higher implementation rate of 89% in patients with stage IVB disease, likely reflecting a better understanding of the necessity of distant metastases.

The median interval from CCRT to chemotherapy initiation was 39 days (range: 1–141 days), compared with 2–4 weeks in previous reports [19, 20, 21]. This delay may be partially attributable to the more advanced disease burden in stage IVB patients as well as to the retrospective study design and physician‐directed discretion in treatment scheduling.

Notably, only one patient experienced a clinically significant delay due to treatment‐related toxicity (Grade 3 enterocolitis), suggesting that CCRT is a generally tolerable approach, even in patients with stage IVB cervical cancer.

### 4.3 Prognosis After CCRT

In this study, the median OS of patients with FIGO stage IVB cervical cancer treated initially with CCRT was 23 months, which this outcome is favorable compared with the previously reported OS of 14.4 months for systemic chemotherapy combined with pelvic irradiation [13]. Importantly, no significant survival differences were observed even among patients with polymetastatic disease or visceral metastases, suggesting that CCRT may confer a potential benefit in subgroups with poor prognosis. An ongoing multicenter retrospective study (JGOG1088S/JROSG22‐1) will further assess the efficacy and safety of whole‐pelvic irradiation in this setting.

In the present cohort of patients with stage IVB cervical cancer, no cases received immune checkpoint inhibitors (ICIs) following CCRT. Recent phase III trials incorporating ICIs have demonstrated a marked improvement in the prognosis of stage IVB cervical cancer [22, 23]. Furthermore, radiotherapy has been shown to enhance immune surveillance by modulating the tumor microenvironment and promoting tumor antigen release [24]. In locally advanced cervical cancer, adding ICIs during and after CCRT improves survival [25]. Our data suggest systemic chemotherapy can be administered safely after CCRT, supporting the feasibility of this sequence. Thus, CCRT followed by ICI‐containing therapy could represent a promising strategy achieving both locoregional control and creating a favorable immunological milieu. Further studies are required to evaluate the combination of CCRT and ICIs for the treatment of stage IVB cervical cancer.

## 5 Limitation

This was a single‐center retrospective observational analysis with a relatively small sample size, which may have limited its statistical power. In addition, treatment selection was not standardized, and potential selection bias could not be excluded, as patients with more advanced disease were more likely to receive chemotherapy or the best supportive care.

## Author Contributions

**Yumi Ishidera:** writing – original draft, investigation, methodology. **Takayoshi Iijima:** writing – review and editing, data curation. **Masahiro Aichi:** formal analysis, writing – review and editing. **Yuki Ogawara:** writing – review and editing, data curation. **Yuichi Imai:** writing – review and editing, data curation. **Madoka Sugiura:** writing – review and editing, data curation. **Masaharu Hata:** supervision. **Etsuko Miyagi:** supervision. **Taichi Mizushima:** writing – original draft, project administration, conceptualization.

## Disclosure

An abstract of this study has been published and the study is scheduled to be presented as a poster at the 2025 Annual Meeting of the Japanese Society of Cancer Therapy, to be held in Yokohama, Japan, on October 18, 2025.

## Ethics Statement

This retrospective study was approved by the Yokohama City University Hospital Institutional Review Board (approval No. F240400003), and the requirement for individual informed consent was waived owing to the retrospective design.

## Consent

The authors have nothing to report.

## Conflicts of Interest

The authors declare no conflicts of interest.

## Supporting information


**Table S1:** Secondary chemotherapy regimens after CCRT.

## Data Availability

The datasets generated and/or analyzed in this study were derived from a single institution with a limited number of cases, which may allow for the identification of individual patients. Therefore, in accordance with the Ethical Guidelines for Medical and Biological Research Involving Human Subjects in Japan, the data are not publicly available. Deidentified data may be provided by the corresponding author upon reasonable request and with approval from the Yokohama City University Hospital Institutional Review Board.
