# Supplementary material for: Efficacy and Safety of Concurrent Chemoradiotherapy as First‐Line Treatment for Stage IVB Cervical Cancer: A Single‐Center Retrospective Observational Study
Source: J Obstet Gynaecol Res. 2025 Oct 29;51(11):e70119. doi: 10.1111/jog.70119 (PMC12571556; doi:10.1111/jog.70119)
Supplement: Supplementary file 1 — Table S1: Secondary chemotherapy regimens after CCRT. [file JOG-51-0-s001.docx]

Supplementary Table 1. Secondary Chemotherapy Regimens After CCRT

| **Chemotherapy (dose)** | **Cycle interval** | n |
| --- | --- | --- |
| Paclitaxel 175 mg/m² + Carboplatin AUC 5 | Every 3 weeks | 20 |
| Docetaxel 70 mg/m² + Carboplatin AUC 5 | Every 3 weeks | 5 |
| Paclitaxel 175 mg/m² + Carboplatin AUC 5 + Bevacizumab 15 mg/kg | Every 3 weeks | 2 |
| Paclitaxel 175 mg/m² + Cisplatin 50 mg/m² + Bevacizumab 15 mg/kg | Every 3 weeks | 2 |
| Irinotecan 60 mg/m², days 1, 8, 15 + Cisplatin 60 mg/m² | Every 3 weeks | 2 |
| Irinotecan 100 mg/m², days 1, 8, 15 | Every 4 weeks | 1 |
| Etoposide 50 mg/day (orally), days 1–21 | Every 4-5 weeks | 1 |
